# Major role of positive selection in the evolution of conservative segments of *Drosophila* proteins

**DOI:** 10.1098/rspb.2012.0776

**Published:** 2012-06-06

**Authors:** Georgii A. Bazykin, Alexey S. Kondrashov

**Affiliations:** 1 Department of Bioengineering and Bioinformatics, Lomonosov Moscow State University, Vorbyevy Gory 1-73, Moscow 119992, Russia; 2 Sector for Molecular Evolution, Institute for Information Transmission Problems of the Russian Academy of Sciences (Kharkevich Institute), Bolshoi Karetny pereulok 19, Moscow 127994, Russia; 3 Life Sciences Institute and Department of Ecology and Evolutionary Biology, University of Michigan, Ann Arbor, MI 48109-2216, USA

**Keywords:** positive selection, negative selection, McDonald–Kreitman test, double substitutions

## Abstract

Slow evolution of conservative segments of coding and non-coding DNA is caused by the action of negative selection, which removes new mutations. However, the mode of selection that affects the few substitutions that do occur within such segments remains unclear. Here, we show that the fraction of allele replacements that were driven by positive selection, and the strength of this selection, is the highest within the conservative segments of *Drosophila* protein-coding genes. The McDonald–Kreitman test, applied to the data on variation in *Drosophila melanogaster* and in *Drosophila simulans*, indicates that within the most conservative protein segments, approximately 72 per cent (approx. 80%) of allele replacements were driven by positive selection, as opposed to only approximately 44 per cent (approx. 53%) at rapidly evolving segments. Data on multiple non-synonymous substitutions at a codon lead to the same conclusion and additionally indicate that positive selection driving allele replacements at conservative sites is the strongest, as it accelerates evolution by a factor of approximately 40, as opposed to a factor of approximately 5 at rapidly evolving sites. Thus, random drift plays only a minor role in the evolution of conservative DNA segments, and those relatively rare allele replacements that occur within such segments are mostly driven by substantial positive selection.

## 1. Introduction

Depending on how the population is located on the fitness landscape, natural selection can be negative or positive. Negative selection operates when the common genotype has the highest fitness, works against rare genotypes and prevents evolution. In contrast, positive selection operates when a rare genotype has the highest fitness, works against the common genotype and facilitates evolution. At any particular moment, the target for negative selection in the genome is much larger than the target for positive selection. As a result, most of the functionally important segments evolve more slowly than the selectively neutral sequence segments [1]. From interspecies sequence comparisons, positive selection can be detected by accelerated evolution [2–4], and it is frequently implied that positive selection plays a larger role in the evolution of rapidly evolving sequence segments [5–11].

However, this conjecture does not follow from any population genetic theory. On the one hand, rapid evolution of some of the sequence segments can be due to pervasive positive selection. On the other hand, if rapidly evolving segments are mostly selectively neutral, which is feasible owing to genome-wide preponderance of negative selection [1], the role of positive selection in their evolution may well be low, compared with that in the evolution of conservative segments, where selective neutrality is likely to be rare owing to a stronger selective constraint. Both situations are possible theoretically.

There were few explicit tests of the conjecture that positive selection is particularly important at rapidly evolving sites, and they led to contradictory results. The fraction of substitutions driven to fixation by positive selection was found to be either indistinguishable among genes with different rates of amino acid evolution [12–15], or somewhat higher in rapidly evolving genes [10]. In contrast, some data hint that the relative role of positive selection may be higher at conserved sites. For example, non-synonymous coding sites have the lowest rate of evolution among all categories of genomic sites (i.e. compared with synonymous, intron, UTR or intergenic sites), but also experience the highest rate of adaptive evolution [16]. A recent analysis of conserved non-coding sites in mice revealed a high fraction of adaptive substitutions, exceeding that found in other categories of sites [17]. In humans, a higher rate of selective sweeps indicative of recurrent positive selection is observed in regions with a higher density of conserved non-coding sites [18]; and in *Drosophila,* clustering of amino acid substitutions at nearby amino acid sites, which is probably caused by epistatic interactions between amino acids, is stronger in more constrained genes [19].

Here, we use complete-genome datasets on between- and within-species genetic variation in *Drosophila* to compare the role of positive selection in the evolution of protein segments of different conservatism. We compare the fraction of the positive selection-driven substitutions, the rate of adaptive evolution and the mean strength of selection associated with the substitutions, between the more and less constrained segments of the proteins. Two different methods of detection of positive selection show that the fraction of substitutions driven by it is the largest in the most conservative protein segments. These results suggest that the evolution of rapidly evolving segments is disproportionally affected by drift, while the rare instances of evolution of conservative segments of protein-coding genes are disproportionally facilitated by positive selection.

## 2. Results

**Figure 1.**
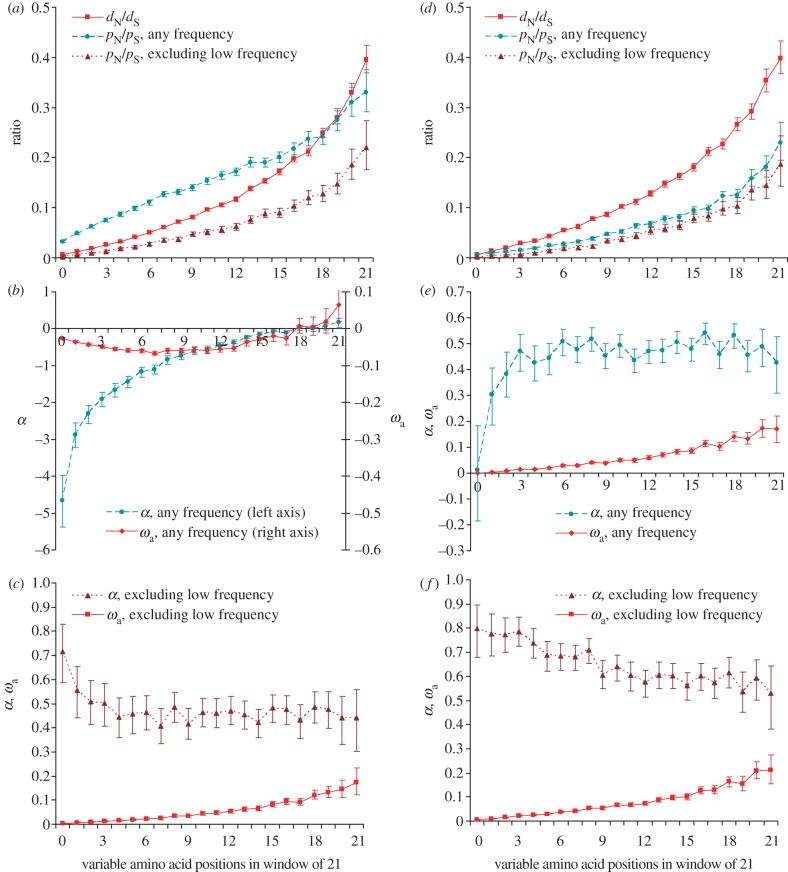
Results of the McDonald–Kreitman test for the protein segments of different conservatism. The McDonald–Kreitman test was applied to the data on variation within the coding sites among (*a*–*c*) 162 individuals of *D. melanogaster* and (*d*–*f*) six individuals of *D. simulans*, and divergence between these species and the *D. yakuba–D. erecta* common ancestor. The sites were subdivided into 22 classes of different conservatism of the protein segments that contain them, in the alignment of their orthologues in seven more distant *Drosophila* species. (*a*,*d*) Ratios of the frequencies of the non-synonymous and synonymous substitutions (*d*_N_*/d*_S_, red squares) and polymorphisms (*p*_N_*/p*_S_); analysis was performed for all polymorphisms (cyan circles) and excluding low-frequency polymorphisms (brown triangles). (*b*,*c*,*e*,*f*) Fraction of positively selected sites **α** and the rate of adaptive non-synonymous substitutions relative to the rate of synonymous substitutions ω_a_ for (*b*,*e*) all polymorphisms and (*c*,*f*) excluding low-frequency polymorphisms. Error bars are 95% CI obtained by non-parametric bootstrapping.

Figure 1*a*–*c* shows the results of the McDonald–Kreitman (MK) test [20–23] performed on divergence data between *Drosophila melanogaster* and the common ancestor of *Drosophila yakuba* and *Drosophila erecta,* and on the variation within 162 *D. melanogaster* genotypes, for coding sites that reside within 21-amino-acid-long sequence segments of different conservatisms. Conservatism of a segment was measured in the species outside of the phylogenetic clade used for the MK test (see §4); therefore, the results of the MK test are not biased by this subdivision.

In the MK test, **α** estimates the fraction of amino acid substitutions that were driven by positive selection; however, this estimate is biased downward by negative selection against segregating deleterious alleles that never reach fixation. When all polymorphisms within *D. melanogaster* are considered, the test consistently produces negative values of **α** (mean **α** = −0.48, 95% CI −0.49 to −0.45), in line with ubiquitous negative selection acting in the polymorphic non-synonymous sites; a higher prevalence of negative selection is observed within slowly evolving segments (figure 1*b*). The confounding effect of negative selection on **α** can be reduced by excluding low-frequency variants [23]. When variants with frequencies below 15 per cent are excluded, **α** increases for segments of any conservatism, suggesting that overall, 0.50 (95% CI 0.48–0.51) of substitutions are driven by positive selection, in line with the current estimates [24–28]. The 9.8 per cent of all sites located in the most conservative segments, however, experience the largest increase: here, **α** reaches approximately 0.72, while it reaches only approximately 0.44 for the sites at rapidly evolving segments (figure 1*c*). A higher **α** in the segments of high conservatism is observed for all cut-off frequency thresholds above approximately 10 per cent (electronic supplementary material, figure S1).

Similar results were obtained using the data on divergence between *Drosophila simulans* and the *D. yakuba–D. erecta* common ancestor, and on variation within six *D. simulans* genotypes (figure 1*d–f*). Here, we get a positive **α** even when all polymorphism is considered: the test produces the overall fraction of positive selection-driven allele replacements **α** = 0.52 (95% CI 0.50–0.53), in agreement with the published estimates [12,21,24,29] (figure 1*e*). The difference from the pattern observed in *D. melanogaster* is due to the differences in the sample size: in a sample of six individuals, even singletons (i.e. alleles observed in only a single individual) often represent high-frequency variants, and their prevalence is shaped by negative selection. Nevertheless, the removal of singletons, again, reverses the dependence of **α** on conservatism. After this correction, the 9.6 per cent of all sites positioned in the most conservative segments have the highest fraction of positively selected substitutions (approx. 80%), while only a marginal increase of **α** is observed at the rapidly evolving segments (figure 1*f*). А somewhat higher **α** observed, after exclusion of the rare variants, in *D. simulans* compared with *D. melanogaster* is consistent with a higher effective population size *N*_e_ in the former [28,30] (but see [31]). A higher impact of excluding low-frequency polymorphisms on **α** at the more conservative segments apparently indicates a higher role of negative selection in shaping the patterns of within-population variation at such segments.

Negative selection affecting synonymous sites, e.g. via translational efficiency, may bias the ratio of synonymous polymorphism to divergence and, consequently, raise **α** upward [28,32,33]. If synonymous selection is stronger in the conservative segments of proteins [34], this bias can lead to an artefactual inference of a higher fraction of positively selected sites in the conservative bins. To control for this effect, we repeated the MK test using the same values of synonymous divergence and polymorphism for each bin, obtained by averaging over all the synonymous sites of the genome. Although some of the differences in **α** between the bins of conservatism could be explained away by differences in synonymous divergence and polymorphism, the overall trend—increase of **α** with conservatism—was robust to this correction both in *D. melanogaster* and in *D. simulans* (see electronic supplementary material, figures S2–S3).

Therefore, higher absolute values of **α** at conservative segments observed after exclusion of low-frequency variants both in *D. melanogaster* and in *D. simulans* indicate that the fraction of positively selected substitutions within such segments is larger. High values of **α** at conservative segments are not associated with a higher overall rate of adaptive evolution: the value of *ω*_a_, which reveals the rate of adaptive non-synonymous divergence relative to the rate of synonymous divergence [35,36], is lower in conservative segments, both in *D. melanogaster* and in *D. simulans* (figure 1). Therefore, the higher value of **α** is due to a lower rate of neutral or weakly selected substitutions, rather than a higher rate of advantageous substitutions, in conservative segments.

To test the robustness of our conclusions, we also estimated **α** and *ω*_a_ for each conservation bin using an extension of the MK test that accounts for the distribution of fitness effects of slightly deleterious mutations [26]. The obtained results (see electronic supplementary material, figure S4) were similar to those obtained in the conventional MK test with the low-frequency polymorphisms excluded.

**Figure 2.**
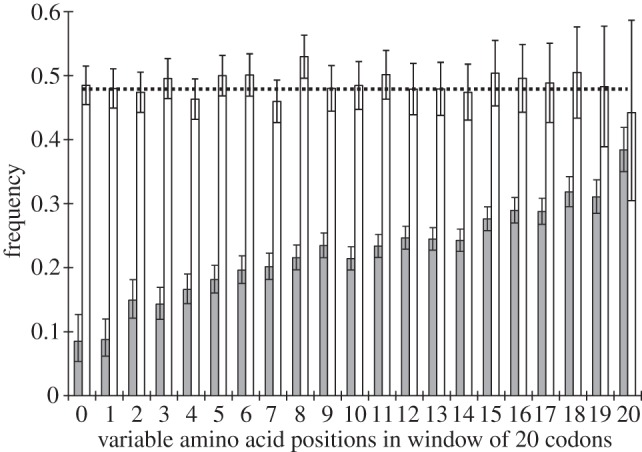
Clumping of pairs of substitutions at a codon site. Fractions of codons, among codons with two substitutions between the *D. simulans–D. sechellia* and the *D. pseudoobscura–D. persimilis* clades, such that these two substitutions occurred in different lineages, for codons residing within segments of different conservatism. Grey bars, non-synonymous substitutions; white bars, synonymous substitutions. The expected value is indicated with a dashed line; error bars are 95% binomial CI.

The MK test does not reveal the strength of positive selection responsible for a positive value of **α**. In order to investigate this strength, we considered the 31 816 codons that underwent two non-synonymous substitutions between *D. simulans* and *Drosophila sechellia*, on the one hand, and *Drosophila pseudoobscura* and *Drosophila persimilis*, on the other hand, with *Drosophila virilis* and *Drosophila mojavensis* serving as an outgroup (pairs of species were used in these comparisons to make sure that the results are not affected by sequencing errors). Positive selection reduces the expected time to a substitution; therefore, at a codon that underwent two non-synonymous substitutions, their clumping indicates positive selection favouring at least the second substitution. This clumping can be revealed by a higher-than-expected occurrence of pairs of substitutions that both occurred in the same lineage [37,38]. Figure 2 shows that this clumping is much stronger at the conservative segments. Within the most conservative class of segments, two non-synonymous substitutions occurred in different lineages only in 0.085 of the codons. Because, without selection, one expects to see this pattern in 0.48 of codons (see §4), this implies that at a fraction **δ** = 0.82 of the two-substitution codons, at least the second non-synonymous substitution was driven by positive selection, in agreement with the result of the MK test. In contrast, at the rapidly evolving segments, two substitutions occurred in different lineages at 0.38 of sites, implying that positive selection operated only at **δ** = 0.21 of such sites. As was the case in the rat–mouse [37] and within-HIV-1 [38] divergence, no clumping was observed at the two-substitution synonymous sites (figure 2).

**Figure 3.**
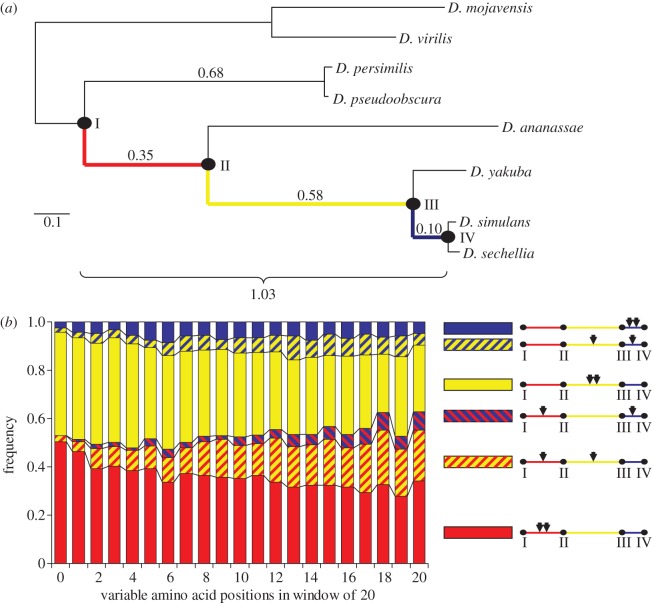
Phylogenetic distribution of pairs of non-synonymous substitutions at a codon site. (*a*) Partial phylogeny of genus *Drosophila*, with lengths of edges in the units of *D*_s_ (adapted from Heger & Ponting [39]); the path to the *D. simulan*s–*D. sechellia* clade (IV) from its common ancestor with the *D. pseudoobscura–D. persimilis* clade (I) is coloured. (*b*) Fractions of codon sites such that the first and the second substitution occurred within particular parts of this path, depending on the conservatism of the segment within which the site resides. For each possible position of the two substitutions relative to the branching-off points of *D. ananassae* (II) and *D. yakuba* (III), the two substitutions at a codon site are shown schematically by arrows at the right panel.

The pairs of substitutions that occurred in the same lineage can be used to estimate the mean expected time to the second substitution, and thus the strength of the positive selection involved. Let us consider the 9118 pairs of non-synonymous substitutions at a codon that both occurred on the path to the *D. simulan*s–*D. sechellia* clade, and take advantage of *Drosophila ananassae* and *D. yakuba* clades that branch off this path (figure 3). For the conservative segments, the fraction of pairs such that the first substitution occurred before branching off of the *D. ananassae* clade (red in figure 3*a*) and the second one occurred soon after this event (yellow in figure 3*a*) is 0.026 (95% CI 0.005–0.073; red- and yellow-striped pattern in figure 3*b*). Because the length, in the units of *d*_S_, of the path to the *D. simulan*s–*D. sechellia* clade is approximately 1.03, this implies that, on average, the second substitution within the pair occurs, after the first substitution, with a lag of approximately 0.025*d*_S_ (i.e. approximately 40 times faster than a selectively neutral substitution). In order to accelerate evolution by a factor of approximately 40, the coefficient of positive selection *s* that drives the second substitution must be such that 4*N*_e_*s* ∼ 40 (see [1], eq. 3.14). In contrast, at the rapidly evolving segments, the fraction of pairs of substitutions that occurred at different sides of the branching-off point of the *D. ananassae* clade is 0.20 (95% CI 0.13–0.30), implying 4*N*_e_*s* ∼ 5 (figure 3*b*).

Moreover, among the conservative codons, where two non-synonymous substitutions occurred on the path to the *D. simulan*s–*D. sechellia* clade, no codons were observed such that the first substitution occurred before *D. ananassae* branching off (red in figure 3*a*), and the second one occurred after *D. yakuba* branching off (blue in figure 3*a*; 95% CI 0–0.031; red- and blue-striped pattern in figure 3*b*). This indicates that the second substitution almost never occurs with a substantial delay and, thus, is almost never neutral. By contrast, at the rapidly evolving segments, such cases comprise 0.076 (95% CI 0.03–0.14) of all pairs (figure 3*b*).

## 3. Discussion

Positive selection is most conspicuous when it causes a particular gene or a sequence segment to evolve very rapidly. Indeed, a commonly used method of detecting positive selection in proteins looks for sequence segments and sites where *d*_N_ > *d*_S_ [2]. In this way, positive selection has been detected, for example, in HIV-1 [40], snake venom [41,42] and semen proteins [43,44]. Thus, although some recent studies demonstrated the importance of positive selection at slowly evolving sequence segments [17], it is still often assumed by default that positive selection plays the largest role in the evolution of the rapidly evolving sites, and that its prevalence in slowly evolving sites is low.

By contrast, our results show that when a conservative segment of a protein accepts an amino acid replacement, which by definition occurs rarely, this replacement is usually driven by strong positive selection. Specifically, the MK test and the co-occurrence of double substitutions in the same lineage concurrently show that the fraction of positively selected non-synonymous substitutions among all non-synonymous substitutions is the highest in the most conservative protein segments; and the clumping of double substitutions along an evolving lineage additionally suggests that the mean selection coefficients involved are high (i.e. that this selection is strong).

Sequencing [45,46] and alignment [45–48] errors may lead to artefactual inference of positive selection. However, sequencing errors are unlikely to affect our results, because in each analysis, we only consider sites such that each variant is observed in more than a single sequence, and identical errors in multiple independent sequences are improbable. As for the alignment, its robustness is expected to be higher in conserved sequences [11,47,48], making our observation of stronger positive selection in the slowly evolving segments conservative. Comparisons of the quality-filtered and unfiltered datasets show that our thorough data filtering also made the results conservative (see §4).

The results of the MK test seem to contrast those obtained in comparisons among different loci, where no [12–15] or a weak positive [10] link between the gene-specific rate of amino acid evolution and values of **α** is observed, suggesting that the observed pattern, or lack thereof, depends on the analysed genomic scale. The probable reason for this difference is that both conservation and the rate of adaptation are likely to vary within a locus [2,49], and using longer windows to assess conservation may blur the signal. The short windows (21 amino acid sites) used here represent the closest we can get to assessing the conservation for individual sites. When even shorter segment lengths were used, the observed patterns in **α*,* *ω*_a_ and **δ** were similar to those presented; however, this increase in resolution came at the cost of increased variance, because for shorter segments, conservation could be assessed with less precision.

In summary, a non-synonymous replacement at a site located within a conservative segment of a protein-coding gene is driven by positive selection substantially more frequently, and this selection is stronger, compared with that typically operating at sites located within rapidly evolving gene segments. The total rate of adaptive allele replacements occurring at rapidly evolving segments is higher than at conservative segments, as revealed by the differences in *ω*_a_; but in the former, adaptive replacements are driven by weaker positive selection and are diluted by a large number of effectively neutral replacements. In contrast, random drift plays almost no role in the evolution of conservative segments of the genome, both coding and non-coding [17], which is almost exclusively driven by strong positive selection. Positive selection-driven allele replacements within generally conservative genome segments may be an important component of adaptive evolution.

## 4. Methods

### (a) Data

Complete genotypes of 162 inbred lines of *D. melanogaster* [50] were obtained from the *Drosophila* Genetic Reference Panel website (http://www.hgsc.bcm.tmc.edu/projects/dgrp/freeze1_July_2010/sequences/). Multiple alignments of genome assemblies of 11 *Drosophila* species [11] to *D. melanogaster* (dm3, BDGP release 5) were obtained from UCSC Genome Bioinformatics Site (http://genome.ucsc.edu). The set of FlyBase canonical splice variants was used to map 13 300 *D. melanogaster* protein-coding genes onto the alignment. Multiple alignment of each coding region was then obtained by joining the aligned segments corresponding to the exons of the FlyBase canonical genes in *D. melanogaster*. The resulting alignments are available at http://makarich.fbb.msu.ru/conservative/.

Complete genotypes of six strains of *D. simulans* [29] were obtained from the *Drosophila* Population Genomics Project website (http://www.dpgp.org/). Since *D. simulans* genotypes were assembled against an earlier *D. melanogaster* reference sequence assembly (dm2, BDGP release 4 [29]), we used the corresponding multiple alignment of 11 *Drosophila* species based on the dm2 *D. melanogaster* reference sequence from UCSC in all analyses involving variation within *D. simulans*. The alignments were processed in the same way as the dm3-based alignments. A total of 13 479 *D. melanogaster* protein-coding genes were mapped onto the alignment.

Only those codon sites in which the reference sequence of each of the 12 species carried a valid codon were considered. Valid codons were defined as those which were aligned and did not contain gaps or non-ACGT characters. We also excluded interspersed repeats and low complexity sequences masked by RepeatMasker [51] and Tandem Repeats Finder [52] with settings as detailed in the UCSC Genome Bioinformatics Site (http://hgdownload.cse.ucsc.edu/goldenPath/dm3/bigZips/README).

### (b) McDonald–Kreitman test

A set of additional data quality filters was applied to each codon site in the multiple alignment prior to the MK test. For each codon site, we required the presence of polymorphism data from 50 per cent of the individuals in the populations in which variation was studied (i.e. 81 individuals for *D. melanogaster,* and three individuals for *D. simulans*). To avoid any possible biases associated with sequencing errors, we took the approach of only making inferences from the codon sites such that each codon state was observed in more than one of the aligned sequences. Specifically, to ensure the quality of the divergence data, only codons matching between *D. yakuba* and *D. erecta* were considered. To ensure the quality of the polarisation, in analyses of variation within *D. melanogaster*, only codons matching between *D. simulans, D. sechellia* and at least one of the non-reference *D. melanogaster* were considered. In analyses of variation within *D. simulans*, only codons matching between *D. melanogaster* and at least one of the non-reference *D. simulans* were considered. Finally, 10 codon sites at the 5′ and the 3′ ends of each gene were excluded from the analysis because their conservatism could not be assessed with certainty. In total, 50.9 per cent of the coding sites in *D. melanogaster,* and 40.9 per cent of the coding sites in *D. simulans,* survived our filtering (see electronic supplementary material, tables S1 and S2). The filtering made our results conservative. Indeed, when no filtering was applied, a more radical contrast between the bins of conservatism was observed, with approximately 86 per cent of the substitutions in the most conservative bin inferred to be under positive selection in *D. melanogaster,* compared with approximately 37 per cent in the least conservative bin (see electronic supplementary material, figure S5); similarly, when all filters were applied except no codon match between *D. yakuba* and *D. erecta* was required, the corresponding values were approximately 86 versus 43 per cent (see electronic supplementary material, figure S6).

The codon sites that survived the filtering were subdivided into 22 bins of conservatism. Conservatism was assigned to each site according to the number (between 0 and 21) of gapless, invariant amino acid positions in alignment of the seven species outside the *melanogaster* subgroup (i.e. *D. ananassae, D. pseudoobscura, D. persimilis, Drosophila willistoni, D. virilis*, *D. mojavensis* and *Drosophila grimshawi*), within a sliding window of 21 amino acid sites spanning the current site, 10 amino acids before it and 10 amino acids after it (see electronic supplementary material, figures S7 and S8). Since only the five species belonging to the *melanogaster* subgroup (*D. melanogaster, D. simulans, D. sechellia, D. yakuba* and *D. erecta*) were involved in the MK test, assessing conservatism outside the *melanogaster* subgroup does not bias the divergence data.

The codon sites belonging to the same bin of conservatism were pooled together across all the loci. An alternative approach would have been to do an MK analysis for each locus separately, and then to combine the results across loci. However, subdividing the data both by locus and by conservatism was impractical: in the *D. melanogaster* dataset, for each particular bin of conservatism, nearly all (99.9%) of the loci had five or fewer polymorphic synonymous sites with derived allele frequency above 0.15, and the vast majority (91.8%) of loci had no such sites. Low values of synonymous polymorphism at a locus are problematic, as they may bias the estimates of alpha [21]. Therefore, we took the popular [16,26,53] alternative strategy of pooling the sites across the genome prior to the analysis.

At each codon site, only non-degenerate nucleotide sites were classified as ‘non-synonymous’, and only fourfold-degenerate nucleotide sites were classified as ‘synonymous’. Non-degenerate and fourfold-degenerate sites were defined as those in which each of the four nucleotides corresponded to a different amino acid, or to the same amino acid, respectively; this condition was required both for the codon observed in the consensus sequence of *D. melanogaster* or *D. simulans,* and for the codon in the *D. yakuba–D.erecta* sequence. Among the non-degenerate and the fourfold-degenerate sites, divergence was defined as the fraction of sites differing between the consensus of *D. melanogaster* (*D. simulans*) sequences and the *D. yakuba–D. erecta* sequence, and polymorphism was defined as the fraction of sites variable within *D. melanogaster* (*D. simulans*). All sites of a given conservatism were pooled together to obtain the values of non-synonymous divergence *d*_N_, synonymous divergence *d*_S_, non-synonymous polymorphism *p*_N_ and synonymous polymorphism *p*_S_. Proportion of amino acid substitutions driven by positive selection **α** was estimated for each bin of conservatism as [21]

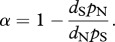

The rate of adaptive non-synonymous substitutions, relative to the rate of synonymous substitutions, was obtained as follows [35]:

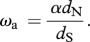

Ninety-five per cent confidence intervals on these values were obtained by bootstrapping individual sites within each bin of conservatism.

Two approaches were used to assess the allele frequencies. For the minor allele frequency, the frequency of the second commonest allele was used. For the derived allele frequency, the frequency of the derived allele was used, with the ancestral variant revealed by *D. simulans* in the analysis of the *D. melanogaster* variation and by *D. melanogaster* in the analysis of the *D. simulans* variation. The results obtained with the two approaches were very similar; the data reported are for the derived allele frequencies. The frequency threshold recommended to reduce the effect of segregating deleterious alleles in the MK test is 15 per cent [23,28,54]. Therefore, we required the presence of an allele in more than 24 out of 162 genotypes of *D. melanogaster* (14.8%), or in more than one out of six genotypes of *D. simulans* (16.7%). Use of higher cut-off values did not affect the results qualitatively (see electronic supplementary material, figure S1).

Our results were robust to the choice of the particular data filters and the details of the analysis. Specifically, if, for each analysed codon, we required data on variation from 100 per cent, rather than 50 per cent, of all individuals, the results remained very similar both for *D. melanogaster* and *D. simulans,* despite reduced sample size. Similar results were also obtained when divergence from *D. simulans–D. sechellia,* rather than from *D. yakuba–D. erecta,* was used in the analysis of variation in the *D. melanogaster* lineage, and when divergence from *D. melanogaster,* rather than from *D. yakuba–D. erecta,* was used in the analysis of variation in the *D. simulans* lineage.

### (c) Double substitutions

We only considered the codon sites where in each of the *D. simulans* and *D. sechellia*, *D. pseudoobscura* and *D. persimilis*, and *D. virilis* and *D. mojavensis* pairs of species, both species carry the same amino acid, in order to make sure that the results are not affected by sequencing errors. Among such sites, we analysed the codon sites in which two non-synonymous substitutions occurred between *D. simulans–D. sechellia* and *D. pseudoobscura*–*D. persimilis;* only those cases were considered where both substitutions are non-synonymous along each of the two possible paths between the two codons [37]. The lineage at which each of the two substitutions occurred was identified using *D. virilis*–*D. mojavensis* as the outgroup; sites where the outgroup did not reveal the ancestral state were not analysed [37]. For the pairs of substitutions that both occurred on the path to the *D. simulans–D. sechellia* clade, the orthologous codons at *D. ananassae* and *D. yakuba* were used to infer the segments of the path at which each of the two substitutions had occurred. Amino-acid-level common ancestry was inferred [37].

Because our analysis of double substitutions involved species spanning the entire phylogeny of *Drosophila*, we could no longer include the current codon site in our procedure for estimation of conservatism, as we did for the MK test. Therefore, for the analysis of double substitutions, we defined 21, rather than 22, bins of conservatism, according to the number of gapless, invariant amino acid positions in the alignment of all 12 *Drosophila* species at 10 amino acids before and 10 amino acids after the current site. The prevalence of double substitutions in codons belonging to each bin of conservatism is shown in electronic supplementary material, figure S9. Exact 95% confidence intervals for the binomial proportions were calculated using the Clopper–Pearson method [55].

### (d) Fraction of positively selected double substitutions

If the substitutions were independent, the expected frequency among the codons with two non-synonymous substitutions between *D. simulans–D. sechellia* and *D. pseudoobscura–D. persimilis* of cases in which one substitution occurred in each of the two lineages (pattern *P*_1_ [37]) is 2*l*_1_*l*_2_ = 0.48, where *l*_1_ = 0.68/1.71 = 0.40 and *l*_2_ = 1.03/1.71 = 0.60 are the proportional lengths of the lineages leading to the *D. simulans–D. sechellia* and the *D. pseudoobscura–D. persimilis* clades, respectively (figure 3*a*). The fraction of double substitutions that were driven to fixation by positive selection **δ** can be calculated as the shortage of pattern *P*_1_ (i.e. excess of double substitutions in the same lineage), compared with the neutral expectations, and equals

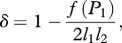

where *f*(*P*_1_) is the fraction of the two-substitution codons in which one substitution occurred in each of the two lineages.

All analyses were done with a set of custom Perl scripts (available upon request).
